# Sufficient and necessary conditions of complete convergence for asymptotically negatively associated random variables

**DOI:** 10.1186/s13660-018-1906-5

**Published:** 2018-11-23

**Authors:** Haiwu Huang, Qingxia Zhang, Xiongtao Wu

**Affiliations:** 1 College of Mathematics and Statistics, Hengyang Normal University, Hengyang, P.R. China; 2 Hunan Provincial Key Laboratory of Intelligent Information Processing and Application, Hengyang, P.R. China; 3 School of Sciences, Southwest Petroleum University, Chengdu, P.R. China

**Keywords:** 60F15, ANA random variables, Complete convergence, Weighed sums, Equivalent conditions

## Abstract

In this investigation, some sufficient and necessary conditions of the complete convergence for weighted sums of asymptotically negatively associated (ANA, in short) random variables are presented without the assumption of identical distribution. As an application of the main results, the Marcinkiewicz–Zygmund type strong law of large numbers based on weighted sums of ANA cases is obtained. The results of this paper extend and generalize some well-known corresponding ones.

## Introduction

The complete convergence is a very important research field in probability limit theory of summation of random variables as well as weighted sums of random variables, which was first introduced by Hsu and Robbins [1] as follows: A sequence $\{ {{X}_{n}};n\ge1 \}$ of random variables converges completely to a constant *λ* if $\sum_{n=1}^{\infty}{P ( \vert {{X}_{n}}-\lambda \vert >\varepsilon )}<\infty$ for all $\varepsilon>0$. In view of the Borel–Cantelli lemma, this implies that ${{X}_{n}}\to \lambda$ almost surely (a.s., in short). Hsu and Robbins [1] proved that the arithmetic means of independent and identically distributed (i.i.d., in short) random variables converges completely to the expected value of the summands, provided the variance is finite. Erdös [2] showed the converse. The Hsu–Robbins–Erdös theorem was generalized in different approaches. One of the most important generalizations was given by Baum and Katz [3] for the following strong law of large numbers.

### Theorem 1.1

*Let* $\frac{1}{2}<\alpha\le1$ *and* $\alpha p\ge1$. *Suppose that* $\{ X,{{X}_{n}};n\ge1 \}$ *is a sequence of i*.*i*.*d*. *random variables with* $E{{X}_{n}}=0$. *Then the following statements are equivalent*:

$$\begin{aligned} (1)&\quad E{{ \vert {X} \vert }^{p}}< \infty ; \\ (2)&\quad \sum_{n=1}^{\infty}{{{n}^{\alpha p-2}}}P \Biggl(\max_{1\le j\le n} \Biggl\vert \sum _{i=1}^{j}{{{X}_{i}}} \Biggr\vert > \varepsilon {{n}^{\alpha}} \Biggr)< \infty\quad \textit{for all } \varepsilon>0 . \end{aligned}$$

Peligrad and Gut [4] extended the result of Baum and Katz [3] for i.i.d. random variables to *ρ̃*-mixing cases.

### Theorem 1.2

*Let* $\frac{1}{2}<\alpha\le1$ *and* $\alpha p>1$. *Suppose that* $\{ X,{{X}_{n}};n\ge1 \}$ *is a sequence of identically distributed* *ρ̃*-*mixing random variables with* $E{{X}_{n}}=0$. *Then the above equations* (1) *and* (2) *are also equivalent*.

However, Peligrad and Gut [4] did not prove whether the result of Baum and Katz [3] for the case $\alpha p=1$ holds for *ρ̃*-mixing random variables. Recently, Cai [5] complemented the result of Peligrad and Gut [4] for the case $\alpha p=1$. For more details about this type of complete convergence theorem, one can refer to Huang et al. [6], Wang and Hu [7], Deng et al. [8], Ding et al. [9], Wu et al. [10] among others.

In the following, some concepts of dependent structures are restated.

### Definition 1.1

Random variables ${{X}_{1}},{{X}_{2}},\ldots ,{{X}_{n}}$ are said to be negatively associated (NA, in short) if, for every pair of disjoint subsets *A* and *B* of $\{ 1,2,\ldots,n \}$ and any real coordinatewise nondecreasing (or nonincreasing) functions ${{f}_{1}}$ and ${{f}_{2}}$,

1.1 $$ \operatorname{Cov} \bigl( {{f}_{1}} ( {{X}_{i}},i\in A ),{{f}_{2}} ( {{X}_{j}},j\in B ) \bigr)\le0, $$

whenever this covariance exists. A sequence $\{ {{X}_{n}};n\ge1 \}$ of random variables is NA if every finite subfamily is NA.

The notion of NA random variables was introduced by Alam and Saxena [11] and carefully studied by Joag-Dev and Proschan [12]. As pointed out and proved by Joag-Dev and Proschan [12], a number of well-known multivariate distributions possess the NA property.

### Definition 1.2

A sequence $\{ {{X}_{n}};n\ge1 \}$ of random variables is called *ρ̃*-mixing if, for some integer $n\ge1$, the mixing coefficient

1.2 $$ \tilde{\rho} ( n )=\sup\sup \biggl\{ \frac{ \vert EXY-EXEY \vert }{\sqrt{\operatorname{Var}X}\sqrt{\operatorname{Var}Y}};X\in {{L}_{2}} \bigl( \sigma(S) \bigr),Y\in{{L}_{2}} \bigl( \sigma(T) \bigr) \biggr\} < 1, $$

where the outside sup is taken over all pairs of nonempty finite sets *S* and *T* of integers such that $\min \{ \vert s-t \vert ,s\in S,t\in T \}\ge n$ and $\sigma ( S )=\sigma \{ {{X}_{i}};i\in S \}$.

### Definition 1.3

A sequence $\{ {{X}_{n}};n\ge1 \}$ of random variables is called asymptotically negatively associated (ANA, in short) if

1.3 $$ {{\rho}^{-}} ( n )=\sup \bigl\{ {{\rho}^{-}} ( S,T ):S,T\subset\mathrm{N}, \operatorname{dist} ( S,T )\ge n \bigr\} \to0\quad \text{as }n\to\infty, $$

where

1.4 $$ {{\rho}^{-}} ( S,T )=0\vee \biggl\{ \frac{\operatorname{Cov} ( f ( {{X}_{i}},i\in S ),g ( {{X}_{j}},j\in T ) )}{\sqrt{\operatorname{Var} ( f ( {{X}_{i}},i\in S ) )\operatorname {Var} ( g ( {{X}_{j}},j\in T ) )}},f,g\in\mathbb {C} \biggr\} , $$

and $\mathbb{C}$ is the set of nondecreasing for every variable functions.

It is obvious that ${{\rho}^{-}} ( n )\le\tilde{\rho} ( n )$, and a sequence of ANA random variables is NA if and only if ${{\rho}^{-}} ( 1 )=0$. Compared with NA and *ρ̃*-mixing, ANA cases define a strictly larger class of random variables (for detailed examples, see [13]). Consequently, extending and improving the convergence theorems for NA and *ρ̃*-mixing random variables to the wider ANA cases is highly desirable in the theory and applications.

In the past decade, many probabilists and statisticians studied and established a series of important results for ANA random variables. For example, see Zhang and Wang [13], Zhang [14, 15] for some moment inequalities of partial sums, the central limit theorems, and the complete convergence, Kim et al. [16] for the strong law of large numbers, Wang and Lu [17] for some moment inequalities of the maximum of partial sums, Wang and Zhang [18] for a Berry–Esséen theorem and the law of the iterated logarithm, Liu and Liu [19] for the moments of the maximum of normed partial sums, Budsaba et al. [20] for the complete convergence for moving average process based on a sequence of ANA and NA random variables, Yuan and Wu [21] for the limiting behavior for ANA random variables under residual Cesàro alpha-integrability assumption, Huang et al. [22] for the complete convergence and the complete moment convergence, Wu and Jiang [23] for the almost sure convergence, and so forth.

Let $\{ {{X}_{n}};n\ge1 \}$ be a sequence of random variables defined on a fixed probability space $( \varOmega, \mathcal {F},P )$, and let $\{{{a}_{n}};n\ge1\}$ be a sequence of real numbers. The probability limit behavior of the maximum weighted sum $\max_{1\le j\le n} \sum_{i=1}^{j}{{{a}_{i}}{{X}_{i}}}$ is very useful in applied probability theory and mathematical statistics. In the theoretical statistical frameworks, many useful linear statistics are based on weighted sums of random samples. For example, least-squares estimators, nonparametric regression function estimators, jackknife estimators, and so on. For that reason, studying the convergence properties for weighted sums of random variables is of much interest.

In this paper, the authors discuss the strong convergence of ANA random variables without identical distributions, and provide some equivalent conditions of Baum–Katz type complete convergence theorem for weighted sums of ANA cases. As an application, the Marcinkiewicz–Zygmund type strong law of large numbers for weighted sums of ANA random variables is also obtained. The main results of this paper extend and improve the known corresponding ones of Peligrad and Gut [4], Cai [5], and Wu and Jiang [23], respectively.

The definition of stochastic domination, which is used frequently throughout this paper, is as follows.

### Definition 1.4

A sequence $\{ {{X}_{n}};n\ge1 \}$ of random variables is said to be stochastically dominated by a random variable *X* if there exists a positive constant *C* such that

1.5 $$ P \bigl( \vert {{X}_{n}} \vert >x \bigr)\le CP \bigl( \vert X \vert >x \bigr) $$

for all $x\ge0$ and $n\ge1$.

Throughout this paper, the symbols $C,{{C}_{1}},{{C}_{2}},\ldots $ will represent generic positive constants which may be different in various places, and ${{a}_{n}}=O ( {{b}_{n}} )$ will mean ${{a}_{n}}\le C{{b}_{n}}$ for all $n\ge1$. $I ( A )$ is the indicator function on the set *A*. $[x]$ denotes the integer part of *x*.

## Main results and proofs

In this section, we will first restate some preliminary lemmas which are useful to proving the main results of this paper.

### Lemma 2.1

*Increasing or decreasing functions defined on disjoint subsets of a sequence of* $\{ {{X}_{n}};n\ge1 \}$ *of ANA random variables with the mixing coefficients* ${{\rho}^{-}}(n)$ *are also ANA random variables with the mixing coefficients not greater than* ${{\rho}^{-}}(n)$.

### Lemma 2.2

(Wang and Lu [17])

*For some positive integers* $n\in \mathbb{N}$ *and* $0\le s<\frac{1}{12}$, *suppose that* $\{ {{X}_{n}};n\ge1 \}$ *is a sequence of ANA random variables with* ${{\rho}^{-}}(n)\le s$, $E{{X}_{n}}=0$, *and* $E{{ \vert {{X}_{n}} \vert }^{2}}<\infty$. *Then there exists a positive constant* $C=C ( 2,n,s )$ *for all* $n\ge1$ *such that*

2.1 $$ E \Biggl(\max_{1\le j\le n} {{ \Biggl\vert \sum _{i=1}^{j}{{{X}_{i}}} \Biggr\vert }^{2}} \Biggr)\le C\sum_{i=1}^{n}{E{{ \vert {{X}_{i}} \vert }^{2}}}. $$

### Lemma 2.3

*For some positive integers* $n\in\mathbb{N}$ *and* $0\le s<\frac{1}{12}$, *suppose that* $\{ {{X}_{n}};n\ge1 \}$ *is a sequence of ANA random variables with* ${{\rho}^{-}}(n)\le s$. *Then there exists a positive constant* *C* *such that*, *for all* $x>0$ *and* $n\ge1$,

2.2 $$ {{ \Bigl( 1-P \Bigl(\max_{1\le i\le n} \vert {{X}_{i}} \vert >x \Bigr) \Bigr)}^{2}}\sum _{i=1}^{n}{P \bigl( \vert {{X}_{i}} \vert >x \bigr)}\le CP \Bigl( \max_{1\le i\le n} \vert {{X}_{i}} \vert >x \Bigr). $$

### Proof

Denote ${{A}_{i}}= ( \vert {{X}_{i}} \vert >x )$ and ${{\alpha}_{n}}=1-P ( \bigcup_{i=1}^{n}{{{A}_{i}}} )=1-P (\max_{1\le i\le n} \vert {{X}_{i}} \vert >x )$. Without loss of generality, assume that ${{\alpha}_{n}}>0$. It follows that $\{ I ( {{X}_{i}}<-x )-EI ( {{X}_{i}}<-x );i\ge1 \}$ and $\{ I ( {{X}_{i}}>x )-EI ( {{X}_{i}}>x );i\ge1 \}$ are two sequences of ANA random variables with the mixing coefficients not greater than ${{\rho}^{-}}(n)\le s$ by Lemma 2.1. Hence, by the ${{C}_{r}}$ inequality and Lemma 2.2, we can have

2.3 $$\begin{aligned} E{{ \Biggl( \sum_{i=1}^{n}{ \bigl( I ( {{A}_{i}} )-EI ( {{A}_{i}} ) \bigr)} \Biggr)}^{2}} \le& 2E{{ \Biggl( \sum_{i=1}^{n}{ \bigl( I ( {{X}_{i}}>x )-EI ( {{X}_{i}}>x ) \bigr)} \Biggr)}^{2}} \\ &{}+2E{{ \Biggl( \sum_{i=1}^{n}{ \bigl( I ( {{X}_{i}}< -x )-EI ( {{X}_{i}}< -x ) \bigr)} \Biggr)}^{2}} \\ \le& C\sum_{i=1}^{n}{P ( {{A}_{i}} )}. \end{aligned}$$

Hence, by Hölder’s inequality and (2.3), we also have that

2.4 $$\begin{aligned} \sum_{i=1}^{n}{P ( {{A}_{i}} )} =&\sum_{i=1}^{n}{P \Biggl( {{A}_{i}}\bigcup_{j=1}^{n}{{{A}_{j}}} \Biggr)} \\ =&\sum_{i=1}^{n}{E \Biggl( I ( {{A}_{i}} )I \Biggl( \bigcup_{j=1}^{n}{{{A}_{j}}} \Biggr) \Biggr)} \\ =&E \Biggl( \sum_{i=1}^{n}{ \bigl( I ( {{A}_{i}} )-EI ( {{A}_{i}} ) \bigr)I \Biggl( \bigcup _{j=1}^{n}{{{A}_{j}}} \Biggr)} \Biggr)+\sum_{i=1}^{n}{P ( {{A}_{i}} )P \Biggl( \bigcup_{j=1}^{n}{{{A}_{j}}} \Biggr)} \\ \le& {{ \Biggl( E{{ \Biggl( \sum_{i=1}^{n}{ \bigl( I ( {{A}_{i}} )-EI ( {{A}_{i}} ) \bigr)} \Biggr)}^{2}}E{{ \Biggl( I \Biggl( \bigcup_{j=1}^{n}{{{A}_{j}}} \Biggr) \Biggr)}^{2}} \Biggr)}^{1/2}}+ ( 1-{{ \alpha}_{n}} )\sum_{i=1}^{n}{P ( {{A}_{i}} )} \\ \le& {{ \Biggl( C ( 1-{{\alpha}_{n}} )\sum _{i=1}^{n}{P ( {{A}_{i}} )} \Biggr)}^{1/2}}+ ( 1-{{\alpha}_{n}} )\sum _{i=1}^{n}{P ( {{A}_{i}} )} \\ \le& \frac{1}{2}{{ \Biggl( \frac{C ( 1-{{\alpha}_{n}} )}{{{\alpha}_{n}}}+{{\alpha}_{n}} \sum_{i=1}^{n}{P ( {{A}_{i}} )} \Biggr)}^{1/2}}+ ( 1-{{\alpha}_{n}} )\sum _{i=1}^{n}{P ( {{A}_{i}} )}. \end{aligned}$$

By reorganizing the above inequality, the desired result (2.2) follows immediately. □

### Lemma 2.4

*Let* $\{ {{X}_{n}};n\ge1 \}$ *be a sequence of random variables which is stochastically dominated by a random variable* *X*. *Then*, *for all* $\alpha>0$, $b>0$, *and* $n\ge1$, *the following statements hold*:

2.5 $$\begin{aligned}& E{{ \vert {{X}_{n}} \vert }^{\alpha}}I \bigl( \vert {{X}_{n}} \vert \le b \bigr)\le{{C}_{1}} \bigl( E{{ \vert X \vert }^{\alpha}}I \bigl( \vert X \vert \le b \bigr)+{{b}^{\alpha}}P \bigl( \vert X \vert >b \bigr) \bigr); \end{aligned}$$

2.6 $$\begin{aligned}& E{{ \vert {{X}_{n}} \vert }^{\alpha}}I \bigl( \vert {{X}_{n}} \vert >b \bigr)\le{{C}_{2}}E{{ \vert X \vert }^{\alpha}}I \bigl( \vert X \vert >b \bigr), \end{aligned}$$

*where* ${{C}_{1}}$ *and* ${{C}_{2}}$ *represent different positive constants*.

Now we state and prove the main results of this paper.

### Theorem 2.1

*Let* $0< p<2$, $\alpha>\frac{1}{2}$, $\alpha p>1$, *and* $0\le s<\frac{1}{12}$. *Suppose that* $\{ {{X}_{n}};n\ge1 \}$ *is a sequence of ANA random variables with the mixing coefficients* ${{\rho}^{-}}(n)\le s$, *which is stochastically dominated by a random variable* *X*. *Assume further that* $E{{X}_{n}}=0$ *if* $1\leq p<2$ *for all* $n\ge1$. *Let* $\{{{a}_{n}};n\ge1\}$ *be a sequence of real numbers such that* $\sum_{i=1}^{n}{{{ \vert {{a}_{i}} \vert }^{2}}}=O ( n )$. *If* $E{{ \vert X \vert }^{p}}<\infty$, *then for all* $\varepsilon>0$

2.7 $$ \sum_{n=1}^{\infty}{{{n}^{\alpha p-2}}P \Biggl(\max_{1\le j\le n} \Biggl\vert \sum _{i=1}^{j}{{{a}_{i}} {{X}_{i}}} \Biggr\vert >\varepsilon{{n}^{\alpha}} \Biggr)}< \infty. $$

### Proof of Theorem 2.1

The proof is primarily inspired by Wang and Wu [24]. Without loss of generality, assume that ${{a}_{n}}\ge0$ for all $n\ge1$. For all $0<\gamma\le2$,

$$ \frac{1}{n}\sum_{i=1}^{n}{{{ \vert {{a}_{i}} \vert }^{\gamma}}}\le {{ \Biggl( \frac{1}{n} \sum_{i=1}^{n}{{{ \vert {{a}_{i}} \vert }^{2}}} \Biggr)}^{{\gamma}/{2} }}, $$

which together with $\sum_{i=1}^{n}{{{ \vert {{a}_{i}} \vert }^{2}}}=O ( n )$ implies that

2.8 $$ \sum_{i=1}^{n}{{{ \vert {{a}_{i}} \vert }^{\gamma}}}=O ( n )\quad \text{for all }0< \gamma\le2. $$

For all $i\ge1$ and $n\ge1$, define

$$ {{X}_{ni}}=-{{n}^{\alpha}}I \bigl( {{X}_{i}}< -{{n}^{\alpha}} \bigr)+{{X}_{i}}I \bigl( \vert {{X}_{i}} \vert \le{{n}^{\alpha}} \bigr)+{{n}^{\alpha}}I \bigl( {{X}_{i}}>{{n}^{\alpha}} \bigr);\qquad {{Y}_{ni}}={{X}_{i}}-{{X}_{ni}}. $$

Therefore, for fixed $n\geq1$, $\{ {{X}_{ni}}-E{{X}_{ni}};i\ge 1 \}$ is still a sequence of ANA random variables by Lemma 2.1.

For all $\varepsilon>0$, it easily follows that

$$\begin{aligned} \Biggl( \max_{1\le j\le n} \Biggl\vert \sum _{i=1}^{j}{{{a}_{i}} {{X}_{i}}} \Biggr\vert >\varepsilon{{n}^{\alpha}} \Biggr) =& \Biggl( \max _{1\le j\le n} \Biggl\vert \sum_{i=1}^{j}{{{a}_{i}} {{X}_{i}}} \Biggr\vert >\varepsilon{{n}^{\alpha }},\bigcap _{i=1}^{n}{ ( {{X}_{i}}={{X}_{ni}} )} \Biggr) \\ &{}\cup \Biggl( \max_{1\le j\le n} \Biggl\vert \sum _{i=1}^{j}{{{a}_{i}} {{X}_{i}}} \Biggr\vert >\varepsilon{{n}^{\alpha }},\bigcup _{i=1}^{n}{ ( {{X}_{i}} \ne{{X}_{ni}} )} \Biggr) \\ \subset& \Biggl( \max_{1\le j\le n} \Biggl\vert \sum _{i=1}^{j}{{{a}_{i}} {{X}_{ni}}} \Biggr\vert >\varepsilon{{n}^{\alpha}} \Biggr)\cup \Biggl( \bigcup _{i=1}^{n}{ \bigl( \vert {{X}_{i}} \vert >{{n}^{\alpha}} \bigr)} \Biggr), \end{aligned}$$

which implies

$$ P \Biggl( \max_{1\le j\le n} \Biggl\vert \sum _{i=1}^{j}{{{a}_{i}} {{X}_{i}}} \Biggr\vert >\varepsilon{{n}^{\alpha}} \Biggr)\le P \Biggl( \max _{1\le j\le n} \Biggl\vert \sum_{i=1}^{j}{{{a}_{i}} {{X}_{ni}}} \Biggr\vert >\varepsilon{{n}^{\alpha}} \Biggr)+P \Biggl( \bigcup_{i=1}^{n}{ \bigl( \vert {{X}_{i}} \vert >{{n}^{\alpha}} \bigr)} \Biggr). $$

In the following, we will proceed with three cases.

*Case* 1: For $\alpha>\frac{1}{2}$, $\alpha p>1$, and $1< p<2$. Firstly, we will show that

2.9 $$ {{n}^{-\alpha}}\max_{1\le j\le n} \Biggl\vert \sum_{i=1}^{j}{E{{a}_{i}} {{X}_{ni}}} \Biggr\vert \to0 \quad \text{as }n\to\infty. $$

Note that $\vert {{Y}_{ni}} \vert \le \vert {{X}_{i}} \vert I ( \vert {{X}_{i}} \vert >{{n}^{\alpha}} )$ and $E{{X}_{n}}=0$ for all $n\ge1$,

2.10 $$\begin{aligned} {{n}^{-\alpha}}\max_{1\le j\le n} \Biggl\vert \sum _{i=1}^{j}{E{{a}_{i}} {{X}_{ni}}} \Biggr\vert =&{{n}^{-\alpha}} \max_{1\le j\le n} \Biggl\vert \sum_{i=1}^{j}{E{{a}_{i}} {{Y}_{ni}}} \Biggr\vert \\ \le& {{n}^{-\alpha}}\sum_{i=1}^{n}{ \vert {{a}_{i}} \vert E \vert {{X}_{i}} \vert I \bigl( \vert {{X}_{i}} \vert >{{n}^{\alpha}} \bigr)} \\ \le& {{n}^{-\alpha}}\sum_{i=1}^{n}{ \vert {{a}_{i}} \vert E \vert X \vert I \bigl( \vert X \vert >{{n}^{\alpha}} \bigr)} \\ \le& {{n}^{1-\alpha}}E \vert X \vert I \bigl( \vert X \vert >{{n}^{\alpha}} \bigr) \\ \le& C{{n}^{1-\alpha p}}E{{ \vert X \vert }^{p}}\to0 \quad \text{as }n\to\infty. \end{aligned}$$

Hence, for *n* large enough and all $\varepsilon>0$,

2.11 $$ {{n}^{-\alpha}}\max_{1\le j\le n} \Biggl\vert \sum_{i=1}^{j}{E{{a}_{i}} {{X}_{ni}}} \Biggr\vert < \frac{\varepsilon}{2}. $$

To prove (2.7), it suffices to show that

2.12 $$\begin{aligned}& {{I}_{1}}\doteq\sum_{n=1}^{\infty}{{{n}^{\alpha p-2}}}P \Biggl( \max_{1\le j\le n} \Biggl\vert \sum _{i=1}^{j}{{{a}_{i}} ( {{X}_{ni}}-E{{X}_{ni}} )} \Biggr\vert >\frac{\varepsilon{{n}^{\alpha }}}{2} \Biggr)< \infty; \end{aligned}$$

2.13 $$\begin{aligned}& {{I}_{2}}\doteq\sum_{n=1}^{\infty}{{{n}^{\alpha p-2}}}P \Biggl( \bigcup_{i=1}^{n}{ \bigl( \vert {{X}_{i}} \vert >{{n}^{\alpha}} \bigr)} \Biggr)< \infty. \end{aligned}$$

By some standard computations, we can easily have that

2.14 $$\begin{aligned} {{I}_{2}} \le& C\sum_{n=1}^{\infty}{{{n}^{\alpha p-2}}} \sum_{i=1}^{n}{P \bigl( \vert {{X}_{i}} \vert >{{n}^{\alpha}} \bigr)} \\ \leq&C\sum_{j=0}^{\infty}{\sum _{n={{2}^{j}}}^{{{2}^{j+1}}-1}{{{n}^{\alpha p-1}}P \bigl( \vert X \vert >{{n}^{\alpha}} \bigr)}} \\ \le& C\sum_{j=1}^{\infty}{{{2}^{j ( \alpha p-1 )}} {{2}^{j}}P \bigl( \vert X \vert >{{2}^{j\alpha}} \bigr)} \\ \le& C\sum_{j=1}^{\infty}{{{2}^{j\alpha p}} \sum_{k=j}^{\infty }{P \bigl( {{2}^{\alpha k}}< \vert X \vert \le{{2}^{\alpha ( k+1 )}} \bigr)}} \\ \le& C\sum_{k=1}^{\infty}{{{2}^{k\alpha p}}P \bigl( {{2}^{\alpha k}}< \vert X \vert \le{{2}^{\alpha ( k+1 )}} \bigr)} \\ \le& CE{{ \vert X \vert }^{p}}< \infty. \end{aligned}$$

For ${{I}_{1}}$, it follows from the Markov inequality, Lemma 2.2, (2.5) of Lemma 2.4 that

2.15 $$\begin{aligned} {{I}_{1}} \le& C\sum_{n=1}^{\infty}{{{n}^{\alpha p-2-2\alpha }}}E \Biggl( \max_{1\le j\le n} {{ \Biggl\vert \sum _{i=1}^{j}{{{a}_{i}} ( {{X}_{ni}}-E{{X}_{ni}} )} \Biggr\vert }^{2}} \Biggr) \\ \le& C\sum_{n=1}^{\infty}{{{n}^{\alpha p-2-2\alpha}}} \sum_{i=1}^{n}{{{a}_{i}}E{{ \vert {{X}_{ni}}-E{{X}_{ni}} \vert }^{2}}} \\ \le& C\sum_{n=1}^{\infty}{{{n}^{\alpha p-2\alpha-1}}E{{ \vert X \vert }^{2}}I \bigl( \vert X \vert \le{{n}^{\alpha}} \bigr)}+C\sum_{n=1}^{\infty}{{{n}^{\alpha p-1}}P \bigl( \vert X \vert >{{n}^{\alpha}} \bigr)} \\ =& C\sum_{n=1}^{\infty}{{{n}^{\alpha p-2\alpha-1}} \sum_{j=1}^{n}{E{{ \vert X \vert }^{2}}I \bigl( j-1< {{ \vert X \vert }^{1/\alpha}}\le j \bigr)}}+CE{{ \vert X \vert }^{p}} \\ \le& C\sum_{j=1}^{\infty}{E{{ \vert X \vert }^{2}}I \bigl( j-1< {{ \vert X \vert }^{1/\alpha}}\le j \bigr) \sum_{n=j}^{\infty }{{{n}^{\alpha p-2\alpha-1}}}} \\ \le& C\sum_{j=1}^{\infty}{{{j}^{\alpha p-2\alpha}}E{{ \vert X \vert }^{2}}I \bigl( j-1< {{ \vert X \vert }^{1/\alpha}}\le j \bigr)} \\ \le& CE{{ \vert X \vert }^{p}}< \infty. \end{aligned}$$

*Case* 2: For $\alpha>\frac{1}{2}$, $\alpha p>1$, and $p=1$. Note that $\alpha>1$ if $\alpha p>1$. By (2.8) and (2.6) of Lemma 2.4, we have that

2.16 $$\begin{aligned} {{n}^{-\alpha}}\max_{1\le j\le n} \Biggl\vert \sum _{i=1}^{j}{E{{a}_{i}} {{X}_{ni}}} \Biggr\vert \le& {{n}^{-\alpha}}\sum_{i=1}^{n}{{{a}_{i}} \bigl( E \vert {{X}_{i}} \vert I \bigl( \vert {{X}_{i}} \vert >{{n}^{\alpha}} \bigr)+{{n}^{\alpha}}P \bigl( \vert {{X}_{i}} \vert >{{n}^{\alpha}} \bigr) \bigr)} \\ \le& C{{n}^{1-\alpha}}E \vert X \vert I \bigl( \vert X \vert >{{n}^{\alpha}} \bigr)\to0. \end{aligned}$$

Hence, by an argument similar to those in the proofs of (2.14) and (2.15), we also have ${{I}_{1}}< CE \vert X \vert <\infty$ and ${{I}_{2}}< CE \vert X \vert <\infty$.

*Case* 3: For $\alpha>\frac{1}{2}$, $\alpha p>1$, and $0< p<1$. By the Markov inequality, (2.5) of Lemma 2.4, and (2.8), we have that

2.17 $$\begin{aligned} {J_{1}} \doteq&\sum_{n=1}^{\infty}{{{n}^{\alpha p-2}}P \Biggl( \max_{1\le j\le n} \Biggl\vert \sum _{i=1}^{j}{{{a}_{i}} {{X}_{i}}I \bigl( \vert {{X}_{i}} \vert \le {{n}^{\alpha}} \bigr)} \Biggr\vert > \frac{\varepsilon{{n}^{\alpha}}}{2} \Biggr)} \\ \le& C\sum_{n=1}^{\infty}{{{n}^{\alpha p-2-\alpha}} \sum_{i=1}^{n}{{{a}_{i}}E \vert {{X}_{i}} \vert I \bigl( \vert {{X}_{i}} \vert \le{{n}^{\alpha}} \bigr)}} \\ \le& C\sum_{n=1}^{\infty}{{{n}^{\alpha p-2-\alpha}} \sum_{i=1}^{n}{{{a}_{i}} \bigl( E \vert X \vert I \bigl( \vert X \vert \le {{n}^{\alpha}} \bigr)+{{n}^{\alpha}}P \bigl( \vert X \vert >{{n}^{\alpha}} \bigr) \bigr)}} \\ \le& C\sum_{n=1}^{\infty}{{{n}^{\alpha p-1-\alpha}}E \vert X \vert I \bigl( \vert X \vert \le{{n}^{\alpha}} \bigr)}+C\sum _{n=1}^{\infty}{{{n}^{\alpha p-1}}P \bigl( \vert X \vert >{{n}^{\alpha}} \bigr)} \\ \le& C\sum_{n=1}^{\infty}{{{n}^{\alpha p-1-\alpha}} \sum_{j=1}^{n}{E \vert X \vert I \bigl( j-1< {{ \vert X \vert }^{1/\alpha }}\le j \bigr)}}+C\sum _{n=1}^{\infty}{{{n}^{\alpha p-1}}\sum _{j=n}^{\infty}{P \bigl( j< {{ \vert X \vert }^{1/\alpha}}\le j+1 \bigr)}} \\ \le& C\sum_{j=1}^{\infty}{{{j}^{\alpha p-\alpha}}E \vert X \vert I \bigl( {{ ( j-1 )}^{\alpha}}< \vert X \vert \le {{j}^{\alpha}} \bigr)}+C\sum_{j=1}^{\infty}{{{j}^{\alpha p}}P \bigl( {j^{\alpha}}< \vert X \vert \le{{ ( j+1 )}^{\alpha}} \bigr)} \\ \le& C{{ \vert X \vert }^{p}}< \infty. \end{aligned}$$

Similarly, we also have that

2.18 $$\begin{aligned} {J_{2}} \doteq&\sum_{n=1}^{\infty}{{{n}^{\alpha p-2}}P \Biggl( \max_{1\le j\le n} \Biggl\vert \sum _{i=1}^{j}{{{a}_{i}} {{X}_{i}}I \bigl( \vert {{X}_{i}} \vert \ge {{n}^{\alpha}} \bigr)} \Biggr\vert > \frac{\varepsilon{{n}^{\alpha}}}{2} \Biggr)} \\ \le& C\sum_{n=1}^{\infty}{{{n}^{\alpha p-2- ( {\alpha p}/{2} )}}E \Biggl(\max_{1\le j\le n} {{ \Biggl\vert \sum _{i=1}^{j}{{{a}_{i}} {{X}_{i}}I \bigl( \vert {{X}_{i}} \vert >{{n}^{\alpha}} \bigr)} \Biggr\vert }^{{p}/{2} }} \Biggr)} \\ \le& C\sum_{n=1}^{\infty}{{{n}^{ ( {\alpha p}/{2} )-2}} \sum_{i=1}^{n}{a_{i}^{{p}/{2} }E{{ \vert {{X}_{i}} \vert }^{{p}/{2} }}I \bigl( \vert {{X}_{i}} \vert >{{n}^{\alpha}} \bigr)}} \\ \le& C\sum_{n=1}^{\infty}{{{n}^{ ( {\alpha p}/{2} )-1}}E{{ \vert X \vert }^{{p}/{2} }}I \bigl( \vert X \vert >{{n}^{\alpha}} \bigr)} \\ \le& C\sum_{n=1}^{\infty}{{{n}^{ ( {\alpha p}/{2} )-1}} \sum_{j=n}^{\infty}{E{{ \vert X \vert }^{{p}/{2} }}I \bigl( j< {{ \vert X \vert }^{1/\alpha}}\le j+1 \bigr)}} \\ =&C\sum_{j=1}^{\infty}{E{{ \vert X \vert }^{{p}/{2} }}I \bigl( j< {{ \vert X \vert }^{1/\alpha}}\le j+1 \bigr) \sum_{n=1}^{j}{{{n}^{ ( {\alpha p}/{2} )-1}}}} \\ \le& C\sum_{j=1}^{\infty}{{{j}^{ ( {\alpha p}/{2} )}}E{{ \vert X \vert }^{{p}/{2} }}I \bigl( j< {{ \vert X \vert }^{1/\alpha}}\le j+1 \bigr)} \\ \le& C{{ \vert X \vert }^{p}}< \infty. \end{aligned}$$

Hence, the desired result (2.7) can be implied from (2.17) and (2.18) for $\alpha>\frac{1}{2}$, $\alpha p>1$, and $0< p<1$ immediately. The proof of Theorem 2.1 is completed. □

The following theorem provides the necessary condition of complete convergence for weighted sums of ANA random variables.

### Theorem 2.2

*Let* $0< p<2$, $\alpha>\frac{1}{2}$, $\alpha p>1$, *and* $0\le s<\frac{1}{12}$. *Suppose that* $\{ {{X}_{n}};n\ge1 \}$ *is a sequence of ANA random variables with the mixing coefficients* ${{\rho}^{-}} ( n )< s$. *Assume that there exist a random variable* *X* *and some positive constant* ${{C}_{1}}$ *such that* ${{C}_{1}}P ( \vert X \vert >x )\le\inf_{n\ge1} P ( \vert {{X}_{n}} \vert >x )$ *for all* $x\ge0$. *Assume further that* $E{{X}_{n}}=0$ *if* $1\leq p<2$. *Let* $\{ {{a}_{n}};n\ge1\}$ *be a sequence of real numbers such that* $\sum_{i=1}^{n}{{{ \vert {{a}_{i}} \vert }^{2}}}=O ( n )$. *Then* (2.7) *implies* $E{{ \vert X \vert }^{p}}<\infty$ *for all* $\varepsilon>0$.

### Proof of Theorem 2.2

Noting that

$$ \max_{1\le i\le n} \vert {{a}_{i}} {{X}_{i}} \vert \le \max_{1\le j\le n} \Biggl\vert \sum _{i=1}^{j}{{{a}_{i}} {{X}_{i}}} \Biggr\vert +\max_{1\le j\le n} \Biggl\vert \sum _{i=1}^{j-1}{{{a}_{i}} {{X}_{i}}} \Biggr\vert . $$

By (2.7), we have that

2.19 $$ \sum_{n=1}^{\infty}{{{n}^{\alpha p-2}}P \Bigl( \max_{1\le i\le n} \vert {{a}_{i}} {{X}_{i}} \vert >\varepsilon{{n}^{\alpha }} \Bigr)}< \infty. $$

For $\alpha p>1$, it follows that

$$\begin{aligned} P \Bigl(\max_{1\le i\le n} \vert {{a}_{i}} {{X}_{i}} \vert >\varepsilon{{n}^{\alpha}} \Bigr)&\le C{{n}^{\alpha p-1}}P \Bigl(\max_{1\le i\le n} \vert {{a}_{i}} {{X}_{i}} \vert >\varepsilon{{n}^{\alpha}} \Bigr) \\ &\le C\sum_{i=n}^{2n}{{{i}^{\alpha p-2}}P \biggl(\max_{1\le j\le i} \vert {{a}_{j}} {{X}_{j}} \vert >\frac{\varepsilon}{{{2}^{\alpha}}}{{i}^{\alpha}} \biggr)}, \end{aligned}$$

which together with (2.19) and the Kronecker lemma implies that

2.20 $$ P \Bigl(\max_{1\le i\le n} \vert {{a}_{i}} {{X}_{i}} \vert >\varepsilon{{n}^{\alpha}} \Bigr)\to0 \quad \text{as }n\to\infty. $$

Hence, for *n* large enough,

2.21 $$ P \Bigl( \max_{1\le i\le n} \vert {{a}_{i}} {{X}_{i}} \vert >\varepsilon{{n}^{\alpha}} \Bigr)< \frac{1}{2}. $$

By Lemma 2.3, (2.21), and ${{C}_{1}}P ( \vert X \vert >x )\le\inf_{n\ge1} P ( \vert {{X}_{n}} \vert >x )$ for all $x\ge0$, we have that

2.22 $$ nP \bigl( \vert {{a}_{i}}X \vert > \varepsilon{{n}^{\alpha}} \bigr)\le\sum_{i=1}^{n}{P \bigl( \vert {{a}_{i}} {{X}_{i}} \vert > \varepsilon{{n}^{\alpha}} \bigr)}\le CP \Bigl( \max_{1\le i\le n} \vert {{a}_{i}} {{X}_{i}} \vert >\varepsilon{{n}^{\alpha }} \Bigr). $$

Take $\varepsilon=1$. By (2.8) for $\gamma=1$ and some standard computations, we have that

2.23 $$\begin{aligned} \infty >&\sum_{n=1}^{\infty}{{{n}^{\alpha p-2}}P \Bigl( \max_{1\le i\le n} \vert {{a}_{i}} {{X}_{i}} \vert >{{n}^{\alpha }} \Bigr)} \\ \ge& C\sum_{n=1}^{\infty}{{{n}^{\alpha p-1}}P \bigl( \vert {{a}_{i}}X \vert >{{n}^{\alpha}} \bigr)} \\ =&C\sum_{n=1}^{\infty}{{{n}^{\alpha p-1}}P \Biggl( \sum_{i=1}^{n}{ \vert {{a}_{i}}X \vert }>\sum_{i=1}^{n}{{{n}^{\alpha}}} \Biggr)} \\ =&C\sum_{n=1}^{\infty}{{{n}^{\alpha p-1}}P \bigl( \vert X \vert \ge{{n}^{\alpha}} \bigr)} \\ \ge&C \sum_{n=1}^{\infty}{{{n}^{\alpha p-1}} \sum_{j=n}^{\infty }{P \bigl( {{j}^{\alpha}} \leq \vert X \vert < {{ ( j+1 )}^{\alpha}} \bigr)}} \\ =&C\sum_{j=1}^{\infty}{P \bigl( {{j}^{\alpha}}\leq \vert X \vert < {{ ( j+1 )}^{\alpha}} \bigr)\sum _{n=1}^{j}{{{n}^{\alpha p-1}}}} \\ \ge& C\sum_{j=1}^{\infty}{P \bigl( {{j}^{\alpha}}\leq \vert X \vert < {{ ( j+1 )}^{\alpha}} \bigr)\sum _{i=1}^{ [ {{\log }_{2}}j ]}{\sum _{n={{2}^{i-1}}}^{{{2}^{i}}-1}{{{n}^{\alpha p-1}}}}} \\ \ge& C\sum_{j=1}^{\infty}{P \bigl( {{j}^{\alpha}}\leq \vert X \vert < {{ ( j+1 )}^{\alpha}} \bigr)\sum _{i=1}^{ [ {{\log }_{2}}j ]}{{{2}^{i\alpha p}}}} \\ \ge& C\sum_{j=1}^{\infty}{P \bigl( {{j}^{\alpha}}\leq \vert X \vert < {{ ( j+1 )}^{\alpha}} \bigr){{2}^{ [ {{\log}_{2}}j ]\alpha p}}} \\ \ge& C\sum_{j=1}^{\infty}{P \bigl( {{j}^{\alpha}}\leq \vert X \vert < {{ ( j+1 )}^{\alpha}} \bigr){{j}^{\alpha p}}} \\ \ge& CE{{ \vert X \vert }^{p}}. \end{aligned}$$

The proof of Theorem 2.2 is completed. □

The following two theorems treat the case $\alpha p=1$.

### Theorem 2.3

*Let* $\frac{1}{2}<\alpha\le1$ *and* $0\le s<\frac {1}{12}$. *Suppose that* $\{ {{X}_{n}};n\ge1 \}$ *is a sequence of mean zero ANA random variables with the mixing coefficients* ${{\rho}^{-}}(n)\le s$, *which is stochastically dominated by a random variable* *X*. *Let* $\{{{a}_{n}};n\ge1\}$ *be a sequence of real numbers such that* $\sum_{i=1}^{n}{{{ \vert {{a}_{i}} \vert }^{2}}}=O ( n )$. *If* $E{{ \vert X \vert }^{p}}<\infty$, *then for all* $\varepsilon>0$,

2.24 $$ \sum_{n=1}^{\infty}{{{n}^{-1}}P \Biggl( \max_{1\le j\le n} \Biggl\vert \sum _{i=1}^{j}{{{a}_{i}} {{X}_{i}}} \Biggr\vert >\varepsilon {{n}^{\alpha}} \Biggr)}< \infty. $$

### Proof of Theorem 2.3

By applying the same notations as those in the proof of Theorem 2.1, we will first show (2.9). For $\frac {1}{2}<\alpha\le1$, note that $1\le p=\frac{1}{\alpha}<2$ if $\alpha p=1$. Therefore, by (2.6) of Lemma 2.4 and $E{{X}_{n}}=0$, we have that

2.25 $$\begin{aligned} {{n}^{-\alpha}}\max_{1\le j\le n} \Biggl\vert \sum _{i=1}^{j}{E{{a}_{i}} {{X}_{ni}}} \Biggr\vert \leq& {{n}^{-\alpha}} \max_{1\le j\le n} \Biggl\vert \sum_{i=1}^{j}{E{{a}_{i}} {{X}_{i}}I \bigl( \vert {{X}_{i}} \vert \le{{n}^{\alpha}} \bigr)} \Biggr\vert +\sum_{i=1}^{n}{ \vert {{a}_{i}} \vert P \bigl( \vert {{X}_{i}} \vert >{{n}^{\alpha}} \bigr)} \\ \le& {{n}^{-\alpha}}\sum_{i=1}^{n}{ \vert {{a}_{i}} \vert E \vert {{X}_{i}} \vert I \bigl( \vert {{X}_{i}} \vert >{{n}^{\alpha}} \bigr) }+nP \bigl( \vert X \vert >{{n}^{\alpha}} \bigr) \\ \le& 2{{n}^{1-\alpha}}E \vert X \vert I \bigl( \vert X \vert >{{n}^{\alpha}} \bigr) \\ =&2{{n}^{1-\alpha}}E{{ \vert X \vert }^{1/\alpha}} {{ \vert X \vert }^{1- ( 1/\alpha )}}I \bigl( \vert X \vert >{{n}^{\alpha }} \bigr) \\ \le& 2E{{ \vert X \vert }^{p}}I \bigl( \vert X \vert >{{n}^{\alpha}} \bigr)\to0 \quad \text{as }n\to\infty. \end{aligned}$$

The rest of the proof is similar to those of *Case* 1 and *Case* 2 in Theorem 2.1, we also have that ${{I}_{1}}\le CE{{ \vert X \vert }^{p}}<\infty$ and ${{I}_{2}}\le CE{{ \vert X \vert }^{p}}<\infty $. The proof of Theorem 2.3 is completed. □

### Theorem 2.4

*Let* $\frac{1}{2}<\alpha\le1$ *and* $0\le s<\frac {1}{12}$. *Suppose that* $\{ {{X}_{n}};n\ge1 \}$ *is a sequence of mean zero ANA random variables with the mixing coefficients* ${{\rho}^{-}}(n)\le s$. *Assume that there exist a random variable* *X* *and some positive constant* ${{C}_{1}}$ *such that* ${{C}_{1}}P ( \vert X \vert >x )\le\inf_{n\ge1} P ( \vert {{X}_{n}} \vert >x )$ *for all* $x\ge0$. *Let* $\{{{a}_{n}};n\ge1\} $ *be a sequence of real numbers such that* $\sum_{i=1}^{n}{{{ \vert {{a}_{i}} \vert }^{2}}}=O ( n )$. *Then* (2.24) *implies* $E{{ \vert X \vert }^{p}}<\infty$ *for all* $\varepsilon>0$.

### Corollary 2.1

*Under the conditions of Theorem *2.1, *if* (2.7) *holds for all* $\varepsilon>0$, *then*

2.26 $$ \sum_{n=1}^{\infty}{{{n}^{\alpha p-2}}P \Biggl( \max_{j\ge n} \Biggl\vert {{j}^{-\alpha}}\sum _{i=1}^{j}{{{a}_{i}} {{X}_{i}}} \Biggr\vert >\varepsilon \Biggr)}< \infty. $$

### Proof of Corollary 2.1

Inspired by the proof of Theorem 12.1 of Gut [25], we can check that by (2.7), for all $\varepsilon>0$,

$$ \sum_{n=1}^{\infty}{{{n}^{\alpha p-2}}P \Biggl( \max_{j\ge n} \Biggl\vert {{j}^{-\alpha}}\sum _{i=1}^{j}{{{a}_{i}} {{X}_{i}}} \Biggr\vert >\varepsilon \Biggr)}\le C\sum_{n=1}^{\infty}{{{n}^{\alpha p-2}}P \Biggl(\max_{1\le j\le n} \Biggl\vert \sum _{i=1}^{j}{{{a}_{i}} {{X}_{i}}} \Biggr\vert >\varepsilon{{n}^{\alpha}} \Biggr)}< \infty. $$

 □

### Corollary 2.2

*Under the conditions of Theorem *2.1 *or Theorem *2.3,

2.27 $$ \lim_{n\to\infty} \frac{1}{{{n}^{\alpha}}}\sum _{i=0}^{n}{{{a}_{i}} {{X}_{i}}}=0 \quad \textit{a.s.} $$

### Proof of Corollary 2.2

Here, we will only prove (2.27) under the conditions of Theorem 2.1. By (2.7), we have that

2.28 $$\begin{aligned} \infty >& \sum_{n=1}^{\infty}{{{n}^{\alpha p-2}}P \Biggl( \max_{1\le j\le n} \Biggl\vert \sum _{i=1}^{j}{{{a}_{i}} {{X}_{i}}} \Biggr\vert >\varepsilon{{n}^{\alpha}} \Biggr)} \\ =& \sum_{i=0}^{\infty}{\sum _{n={{2}^{i}}}^{{{2}^{i+1}}-1}{{{n}^{\alpha p-2}}}P \Biggl( \max _{1\le j\le n} \Biggl\vert \sum_{i=1}^{j}{{{a}_{i}} {{X}_{i}}} \Biggr\vert >\varepsilon{{n}^{\alpha}} \Biggr)} \\ \geq& \textstyle\begin{cases} \sum_{i=0}^{\infty}{{{ ( {{2}^{i}} )}^{\alpha p-1}}P ( \max_{1\le j\le{{2}^{i}}} \vert \sum_{i=1}^{j}{{{a}_{i}}{{X}_{i}}} \vert >\varepsilon{{2}^{ ( i+1 )\alpha}} )},&\mbox{if } \alpha p\ge2, \\ \sum_{i=0}^{\infty}{{{ ( {{2}^{i+1}} )}^{\alpha p-2}}{{2}^{i}}P ( \max_{1\le j\le{{2}^{i}}} \vert \sum_{i=1}^{j}{{{a}_{i}}{{X}_{i}}} \vert >\varepsilon{{2}^{ ( i+1 )\alpha}} )},&\mbox{if } 1< \alpha p< 2 \end{cases}\displaystyle \\ \geq& \textstyle\begin{cases} \sum_{i=0}^{\infty}{P ( \max_{1\le j\le{{2}^{i}}} \vert \sum_{i=1}^{j}{{{a}_{i}}{{X}_{i}}} \vert >\varepsilon {{2}^{ ( i+1 )\alpha}} )},&\mbox{if } \alpha p\ge2, \\ \frac{1}{2}\sum_{i=0}^{\infty}{P ( \max_{1\le j\le {{2}^{i}}} \vert \sum_{i=1}^{j}{{{a}_{i}}{{X}_{i}}} \vert >\varepsilon{{2}^{ ( i+1 )\alpha}} )},&\mbox{if } 1< \alpha p< 2. \end{cases}\displaystyle \end{aligned}$$

In view of the Borel–Cantelli lemma, we also have that

2.29 $$ \lim_{i\to\infty} \frac{\max_{1\le j\le {{2}^{i}}} \vert \sum_{i=1}^{j}{{{a}_{i}}{{X}_{i}}} \vert }{{{2}^{ ( i+1 )\alpha}}}=0 \quad \text{a.s.} $$

For all positive integers *n*, there exists a nonnegative integer ${{i}_{0}}$ such that ${{2}^{{{i}_{0}}-1}}\le n<{{2}^{{{i}_{0}}}}$. Thus

2.30 $$ \frac{1}{{{n}^{\alpha}}}\sum_{i=0}^{n}{{{a}_{i}} {{X}_{i}}}\le \max_{{{2}^{{{i}_{0}}-1}}\le n\le{{2}^{{{i}_{0}}}}} \frac {1}{{{n}^{\alpha}}}\sum _{i=0}^{n}{{{a}_{i}} {{X}_{i}}}\le{{2}^{\alpha }}\frac{\max_{1\le j\le{{2}^{{{i}_{0}}}}} \vert \sum_{i=1}^{j}{{{a}_{i}}{{X}_{i}}} \vert }{{{2}^{ ( {{i}_{0}}+1 )\alpha}}}\to0 \quad \text{a.s.}, $$

which implies

$$ \lim_{n\to\infty} \frac{1}{{{n}^{\alpha}}}\sum _{i=0}^{n}{{{a}_{i}} {{X}_{i}}}=0 \quad \text{a.s.} $$

The proof of Corollary 2.2 is completed. □

### Remark 2.1

Taking ${{a}_{n}}=1$ for all $n\ge1$ in Theorems 2.1–2.4 above, we can also obtain the Baum and Katz type complete convergence theorem for ANA random variables under the cases of $0< p<2$, $\alpha>\frac{1}{2}$, $\alpha p>1$ and $\frac{1}{2}<\alpha \le1$, $\alpha p=1$, respectively. Since ANA random variables include *ρ̃*-mixing random variables and NA random variables, the main results of this paper also hold for *ρ̃*-mixing and NA cases. Hence, Theorems 2.1–2.4 extend the corresponding ones of Peligrad and Gut [4] and Cai [5] to the weighted sums.

### Remark 2.2

Wu and Jiang [23] also investigated the almost sure convergence for identically distributed ANA random variables and obtained the Marcinkiewicz–Zygmund type strong law of large numbers under $E{{ \vert X \vert }^{p}}<\infty$ for $0< p<2$. Compared with their result, it is worth pointing out that we establish some much stronger convergence results for weighted sums of ANA random variables without the assumption of identical distribution, which can imply the corresponding one of Wu and Jiang [23].

## References

[1] Hsu P.L., Robbins H. (1947). Complete convergence and the law of large numbers. Proc. Natl. Acad. Sci. USA.

[2] Erdös P. (1949). On a theorem of Hsu and Robbins. Ann. Math. Stat..

[3] Baum L.E., Katz M. (1965). Convergence rates in the law of large numbers. Trans. Am. Math. Soc..

[4] Peligrad M., Gut A. (1999). Almost-sure results for a class of dependent random variables. J. Theor. Probab..

[5] Cai G.H. (2006). Strong law of large numbers for ${{\rho}^{*}}$-mixing sequences with different distributions. Discrete Dyn. Nat. Soc..

[6] Huang H.W., Wang D.C., Wu Q.Y., Zhang Q.X. (2011). A note on the complete convergence for sequences of pairwise NQD random variables. J. Inequal. Appl..

[7] Wang X.J., Hu S.H. (2014). Complete convergence and complete moment convergence for martingale difference sequence. Acta Math. Sin. Engl. Ser..

[8] Deng X., Ge M.M., Wang X.J., Liu Y.F., Zhou Y. (2014). Complete convergence for weighted sums of a class of random variables. Filomat.

[9] Ding Y., Wu Y., Ma S.L., Tao X.R., Wang X.J. (2017). Complete convergence and complete moment convergence for widely orthant dependent random variables. Commun. Stat., Theory Methods.

[10] Wu Y., Wang X.J., Hu S.H. (2017). Complete moment convergence for weighted sums of weakly dependent random variables and its application in nonparametric regression model. Stat. Probab. Lett..

[11] Alam K., Saxena K.M.L. (1981). Positive dependence in multivariate distributions. Commun. Stat., Theory Methods.

[12] Joag-Dev K., Proschan F. (1983). Negative association of random variables with applications. Ann. Stat..

[13] Zhang L.X., Wang X.Y. (1999). Convergence rates in the strong laws of asymptotically negatively associated random fields. Appl. Math. J. Chin. Univ. Ser. B.

[14] Zhang L.X. (2000). A functional central limit theorem for asymptotically negatively dependent random fields. Acta Math. Hung..

[15] Zhang L.X. (2000). Central limit theorems for asymptotically negative dependent random field. Acta Math. Sci. Ser. B Engl. Ed..

[16] Kim T.S., Ko M.H., Lee I.H. (2004). On the strong laws for asymptotically almost negatively associated random variables. Rocky Mt. J. Math..

[17] Wang J.F., Lu F.B. (2006). Inequalities of maximum partial sums and weak convergence for a class of weak dependent random variables. Acta Math. Sci. Ser. B Engl. Ed..

[18] Wang J.F., Zhang L.X. (2007). A Berry–Esséen theorem and a law of the iterated logarithm for asymptotically negatively associated sequences. Acta Math. Sci. Ser. B Engl. Ed..

[19] Liu X.D., Liu J.X. (2009). Moments of the maximum of normed partial sums of ${{\rho }^{-}}$-mixing random variables. Appl. Math. J. Chin. Univ. Ser. B.

[20] Budsaba K., Chen P.Y., Volodin A. (2007). Limiting behavior of moving average processes based on a sequence of ${{\rho}^{-}}$-mixing random variables. Thail. Stat..

[21] Yuan D.M., Wu X.S. (2010). Limiting behavior of the maximum of the partial sum for asymptotically negatively associated random variables under residual Cesàro alpha-integrability assumption. J. Stat. Plan. Inference.

[22] Huang H.W., Peng J.Y., Wu X.T., Wang B. (2016). Complete convergence and complete moment convergence for arrays of rowwise ANA random variables. J. Inequal. Appl..

[23] Wu Q.Y., Jiang Y.Y. (2018). Some limiting behavior for asymptotically negatively associated random variables. Probab. Eng. Inf. Sci..

[24] Wang X.J., Wu Y. (2017). On complete convergence and complete moment convergence for a class of random variables. J. Korean Math. Soc..

[25] Gut A. (2005). Probability: A Graduate Course.

